# Supplementary material for: Interfacing Digital Microfluidics with Ambient Mass Spectrometry Using SU-8 as Dielectric Layer
Source: Micromachines (Basel). 2018 Dec 8;9(12):649. doi: 10.3390/mi9120649 (PMC6316065; doi:10.3390/mi9120649)
Supplement: Supplementary file 1 [file micromachines-09-00649-s001.zip › micromachines-401797-SI.pdf]

Supplementary Materials: Interfacing Digital Microfluidics with Ambient Mass Spectrometry Using SU-8 as Dielectric Layer

Gowtham Sathyanarayanan, Markus Haapala and Tiina Sikanen

1. Schematic Presentation of the DAPPI-MS Setup

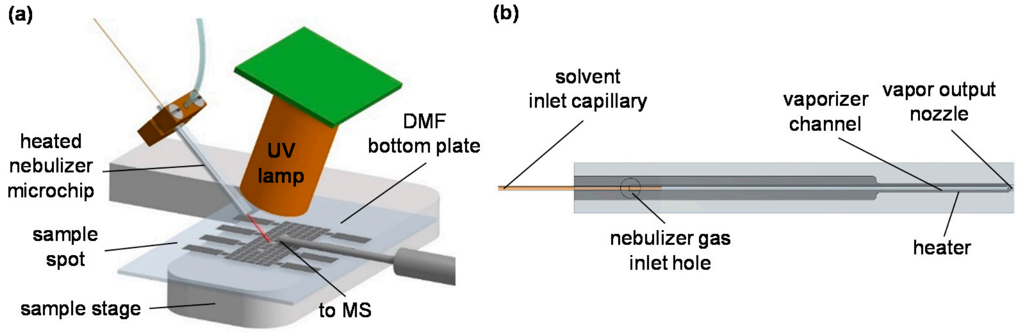

Figure S1. Schematic illustrations of (**a**) DAPPI-MS analysis of samples precipitated onto the DMF bottom plate, and (**b**) the key functions of the heated nebulizer chip.

2. Characterization of the SU-8 and OrmoComp Dielectric Layers

The dielectric properties of SU-8 were tested and compared to those of OrmoComp (a negative tone inorganic-organic hybrid polymer) using DMF driving voltages of 100–140 V_{rms} at 10 kHz. The OrmoComp dielectric layers (8 μm) were spin coated (6000 rpm, 30 s) by increasing the spinning speed in a stepwise manner (at intervals of 1000 rpm per every 6 s), prebaked at 90 °C for 10 min, and flood exposed under UV for 4 s (5000-EC Series UV flood lamp; Dymax corporation, Torrington, CT, USA; nominal intensity 225 mW/cm^2). Otherwise, the chip fabrication process was identical to that of SU-8 as described in the manuscript. The differences in terms of dielectric breakdown tendency between SU-8 and OrmoComp dielectric layers are illustrated in Figure S2. The defects caused by application of the DMF voltages are clearly visible on OrmoComp, whereas SU-8 remains undamaged even after prolonged use.

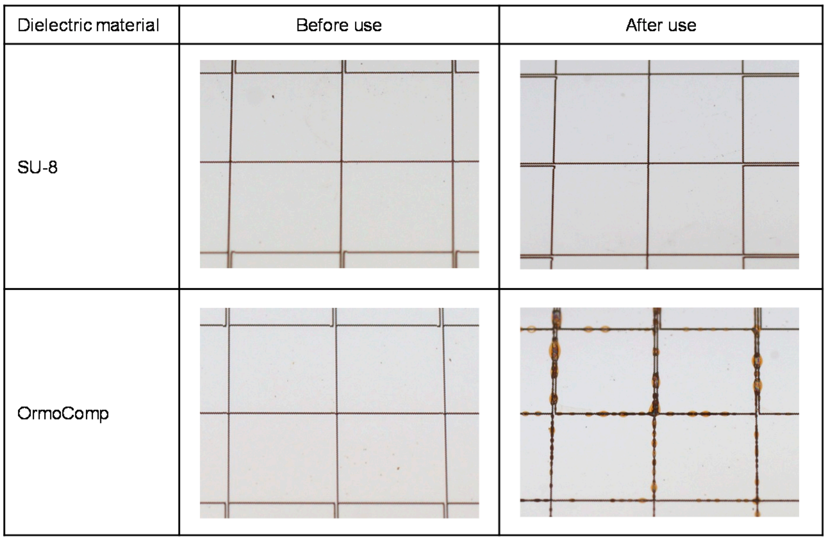

Figure S2. Comparison of SU-8 and Ormocomp dielectric layers before and after use (in DMF, driving voltages (100–140 V_{rms} at 10 kHz).

3. Comparison of the Back Spectra of SU-8 and Fluoropolymer

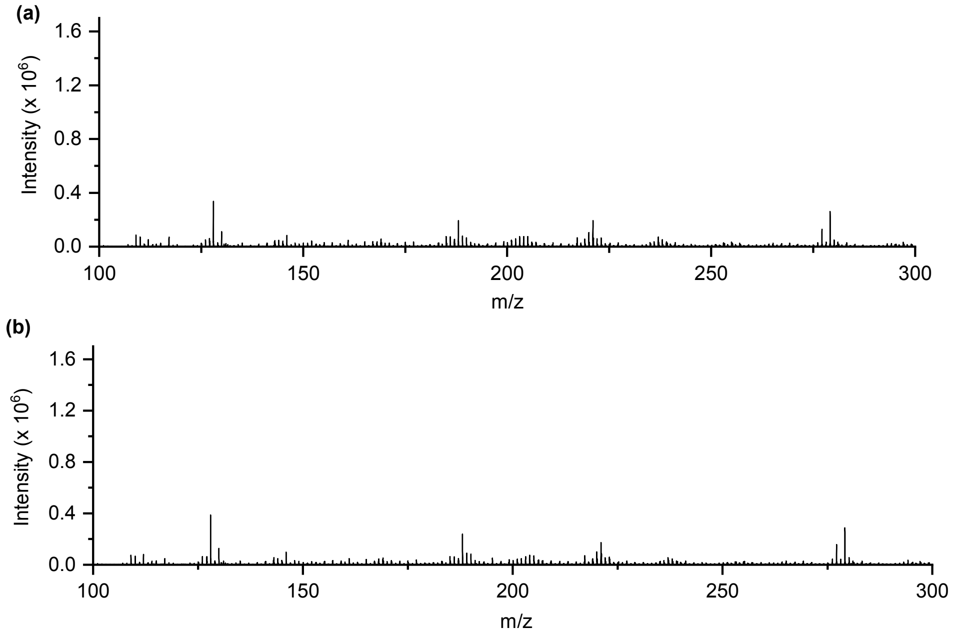

Figure S3. Background spectra measured from (**a**) fluoropolymer coating and (**b**) SU-8 surface.

4. Characterization of the Reuse Possibility of Hydrophilic SU-8 Spots for Repeated DAPPI-MS Analyses

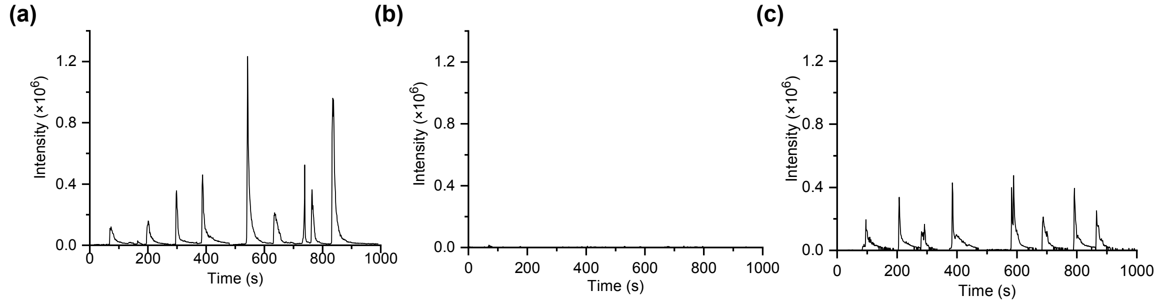

Figure S4. Extracted ion chromatograms corresponding to protonated testosterone ion ($[M + H]^+$ = 289.216) measured from eight parallel SU-8 spots following (**a**) first application of 70 pmol testosterone in methanol-water (1:1) per spot (fresh plate), (**b**) washing of the spots with methanol and water (cleaned plate), and (**c**) re-application of 70 pmol testosterone (cleaned and reused plate).
